# Investigation of Quiet Quitting and Burnout Levels Among Physical Therapy and Rehabilitation Students: A Descriptive and Correlational Study

**DOI:** 10.3390/healthcare14203359

**Published:** 2026-10-09

**Authors:** Ramazan Cihad Yilmaz, Dilan Demirtas Karaoba, Busra Candiri Senol, Alper Percin

**Affiliations:** 1 Department of Physiotherapy and Rehabilitation, Faculty of Health Sciences, Iğdır University, 76000 Iğdır, Türkiye; dilandemirtas92@gmail.com; 2 Department of Physiotherapy and Rehabilitation, Faculty of Health Sciences, İnonu University, 44280 Malatya, Türkiye; busracandiri@gmail.com; 3 Department of Therapy and Rehabilitation, Şiran Dursun Keleş Vocational School of Health Services, Gümüşhane University, Şiran, 29700 Gümüşhane, Türkiye; alperpercin@yahoo.com

**Keywords:** burnout, health professions education, physiotherapy students, quiet quitting

## Abstract

**Background/Objectives**: Quiet quitting is characterized by reduced commitment and reluctance to exceed expected role responsibilities, whereas burnout reflects the depletion of emotional resources due to work and educational demands. This study aimed to examine the relationship between quiet quitting and burnout among Physical Therapy and Rehabilitation (PTR) students and to determine whether these variables differ by gender, grade level, reason for choosing the program, and program satisfaction. **Methods**: A total of 225 students participated in this descriptive and cross-sectional study. Data were collected using the Personal Information Form, the Quiet Quitting Scale (QQS), and the Maslach Burnout Inventory—Student Form (MBI-SF). The data were analyzed using nonparametric statistical methods with the SPSS 27.0 software. **Results**: The mean QQS total score was 48.14 ± 9.80. The mean scores for the MBI-SF dimensions were 14.27 ± 4.66 for Exhaustion, 9.89 ± 3.65 for Cynicism, and 11.28 ± 2.98 for Professional Efficacy. QQS scores differed significantly according to grade level and reason for choosing the programme, whereas gender differences were observed only in the Self-Protection subscale (*p* < 0.05). MBI-SF dimensions did not differ according to gender, grade level, reason for choosing the programme, or programme satisfaction (*p* > 0.05). “Exhaustion” and “Cynicism” were positively correlated with QQS subscale and total scores (r = 0.307–0.639, all *p* < 0.001). The MBI-SF “Professional Efficacy” subscale showed a weak correlation with QQS “Lack of Motivation” (r = 0.143, *p* = 0.032). **Conclusions**: Quiet quitting and burnout were significantly associated among PTR students, with the strength of this relationship varying across burnout dimensions. These findings extend the limited evidence on these constructs in physiotherapy education and support further investigation of their academic and psychosocial context.

## 1. Introduction

Quiet quitting is characterised by an individual’s fulfilment of only the fundamental responsibilities expected of them, a reduction in their voluntary engagement in the role, and a decline in their psychological commitment [1]. This concept is more associated with a decline in motivation, avoidance of extra effort and psychological withdrawal from the process, rather than with physical separation [2]. The shift in expectations regarding work life in the post-pandemic era, the growing importance of the concept of work–life balance, and Generation Z’s different attitudes toward professional life have contributed to “quiet quitting” becoming a more visible phenomenon [3]. Although the concept of ‘quiet quitting’ has primarily been discussed in the context of working life [4], it has also recently been examined in relation to the academic engagement and attitudes of higher education students towards their learning processes [1,5]. Academic quiet quitting is seen as a reflection of the fact that students only fulfil their basic academic obligations, choosing to participate less in the learning process and becoming less engaged academically [5].

The experience of a university education can affect students’ academic engagement and motivation due to factors such as heavy workloads, pressure to perform well, uncertainty about the future and numerous psychosocial stressors [6]. Particularly for health sciences students, the intensity of theoretical courses, the responsibilities associated with clinical practice, and the expectation of professional competence make this process more complex [7]. It has been reported that some students have experienced a decline in their interest in academic activities, have struggled to actively participate in learning, and have focused solely on meeting the mandatory requirements under these circumstances [8].

The students of Physical Therapy and Rehabilitation (PTR) not only acquire theoretical knowledge throughout their education but also play an active role in clinical practice, interact directly with patients, and take on professional responsibilities at an early stage. It has been shown that during clinical practice, students may experience significant levels of stress due to academic performance pressure, patient safety and care responsibilities, and concerns about professional competence [9,10]. It has also been reported that rising stress levels may negatively affect not only academic achievement but also students’ psychological well-being, career motivation, and engagement in the learning process [11]. This situation makes it meaningful to examine the tendencies toward quiet quitting among the students of PTR, especially in the clinical training phase.

Burnout, one of the key factors that may contribute to students’ psychological disengagement from academic processes, is known as a psychological syndrome characterized by emotional exhaustion, depersonalization, and a diminished sense of personal accomplishment [12]. It has been reported that the demanding academic requirements and clinical workload among health sciences students can increase their levels of burnout [6,7]. Previous studies of physiotherapy students have shown that burnout is associated with higher stress levels, lower psychological well-being, hopelessness, and decreased academic engagement [11,13]. Given factors such as loss of motivation, psychological withdrawal, and a decline in academic engagement associated with burnout, it is reasonable to predict that tendencies toward “quiet quitting” may be linked to students’ levels of burnout.

It is evident that studies on the concept of “quiet quitting” have largely focused on the working population in the literature [14,15]. There is a lack of research on the tendency of students, especially those in health sciences, to engage in “quiet quitting” [1]. To the best of our knowledge, no study has been found that evaluates the relationship between tendencies towards quiet quitting and burnout levels among PTR students. This study aimed to identify tendencies towards quiet quitting and levels of burnout among PTR students, and to assess the relationship between these two factors.

Research Questions

What is the level of quiet quitting among PTR students?

What are the levels of the burnout dimensions among PTR students?

Do tendencies toward quiet quitting and levels of burnout vary by gender, grade level, reason for choosing a major, and satisfaction with the major?

Is there a significant relationship between tendencies toward quiet quitting and levels of burnout?

## 2. Materials and Methods

### 2.1. Study Design

A descriptive and correlational research model was used to examine quiet quitting tendencies and burnout levels among the students of PTR in this study.

### 2.2. Population and Sample

The study included 294 undergraduate students enrolled in the Department of PTR at Igdir University during the 2025–26 academic year. Data was collected between 1 and 30 April 2026. The study did not involve sample selection; instead, it aimed to cover the entire population. All students enrolled in the department were informed face-to-face by a faculty member from the research team about the study, the eligibility criteria, and the fact that participation was voluntary. During this briefing, students were asked whether they had any chronic medical conditions diagnosed based on their own self-reports. And the survey participation link was sent to students who met the inclusion criteria and wanted to participate voluntarily. A total of 225 students who voluntarily agreed to participate in the study and who were 18 years of age or older and had completed the data collection instruments in full were included in the study. The participation rate was 76.5%. The study included individuals who were 18 years of age or older, in healthy condition, free of any chronic illnesses, and who voluntarily agreed to participate and provided their informed consent online (via Google Forms). Subjects who were not enrolled in the relevant department, being under the age of 18, declined to participate or did not provide online informed consent, having chronic medical conditions, having physical or mental limitations that could prevent their participation were excluded from the study.

### 2.3. Data Collection Tools

Data were collected using the Personal Information Form, the Quiet Quitting Scale (QQS), and the Maslach Burnout Inventory—Student Form (MBI-SF).

#### 2.3.1. The Personnel Information Form

This form contains questions designed to determine students’ age, gender, grade level, reasons for choosing the department of PTR, and their level of satisfaction with the program [1].

#### 2.3.2. Quiet Quitting Scale (QQS)

The QQS was developed by Savaş and Turan in 2023. The scale assesses students’ quiet quitting tendencies in the academic context. The scale consists of 16 items across 4 subdimensions (Decreased Commitment, Lack of Motivation, Socialization, and Self-Protection). The scale is a 5-point Likert scale (1: Strongly disagree—5: Strongly agree). The lowest score on the scale is 16, and the highest is 80 (16–29 is very low, 30–42 is low, 43–55 is moderate, 56–68 is high, and 69–80 is very high). There are no items subject to reverse coding. Higher scores on the QQS indicate a greater tendency toward quiet quitting. The Cronbach’s alpha coefficient for the QQS has been reported as 0.91 [16].

#### 2.3.3. Maslach Burnout Inventory—Student Form (MBI-SF)

This scale was developed by Schaufeli et al. (2002) to assess levels of burnout among college students [17]. Although the original version of the MBI-SF consists of 16 items, the Turkish adaptation by Çapri et al. (2011) consists of 13 items and three subdimensions: Exhaustion (5 items), Cynicism (4 items), and Professional Efficacy (4 items). The scale is rated as follows: “1: Never,” “2: Sometimes,” “3: Usually,” “4: Most of the time,” and “5: Always.” The items in the Exhaustion dimension of the MBI-SF reflect fatigue and strain resulting from the workload of classes, while the items in the Cynicism subdimension reveal the mental detachment students develop toward their classes. The items in the Professional Efficacy subscale assess students’ perceptions of their academic performance. The sub-dimensions of the scale are interpreted separately. The subscales of Exhaustion and Cynicism also indicate that burnout increases as scores rise. A lower score on the Professional Efficacy subscale indicates increased burnout [18].

### 2.4. Data Collection and Ethics

The data was collected via “Google Forms” from students who met the inclusion criteria. To prevent duplicate submissions, the Google Forms settings were configured to allow only one response per user account. Before the data collection process began, the students were informed about the study and were included on a voluntary basis. Students who agreed to participate in the study were informed about their participation by reading the informational text on the first page of the online form. Participants were informed that the data collected from the study would be used solely for scientific research and that they could withdraw from the study at any time. Students who accept to participate in the study were directed to the data collection form after they had approved the online informed consent form. Data was collected anonymously to ensure the privacy of the participants. Ethical approval for this study was obtained from the Iğdır University Ethics Committee for Non-Interventional Clinical Research (Meeting/Decision No.: 25/11, Date: 25 February 2026). The data collection process was conducted in accordance with the Declaration of Helsinki.

### 2.5. Data Analysis

The statistical analysis of the data was performed using SPSS version 27.0 (IBM Corp., Armonk, NY, USA). The mean and standard deviation, minimum and maximum values, median, and interquartile range (25th–75th percentiles) were used to describe continuous variables. Categorical variables were presented as numbers and percentages. The normality of the data was assessed using the Kolmogorov–Smirnov test. Since the data did not follow a normal distribution when comparing scores on the QQS and the MBI-SF by gender, grade level, reason for choice, and level of satisfaction, the Mann–Whitney U test and the Kruskal–Wallis test were used. When a statistically significant overall difference was detected in the Kruskal–Wallis test, Bonferroni-adjusted pairwise comparisons were performed. The predefined group comparisons were considered primary analyses, whereas the correlation analyses were considered exploratory. Effect sizes were calculated to quantify the magnitude of the observed differences. For Mann–Whitney U tests, the effect size was calculated as (r = |Z|/√N), whereas epsilon squared (ε^2^) was calculated for Kruskal–Wallis H tests. Since the data did not follow a normal distribution, the Spearman correlation test was used to analyze the relationship between MBI-SF and the QQS. A correlation coefficient (r) > 0.8 was interpreted as very strong, 0.6–0.8 as strong, 0.4–0.6 as moderate, 0.2–0.4 as weak, and <0.2 as very weak. *p* < 0.05 was considered statistically significant.

## 3. Results

A total of 294 students were informed about the study. The 225 university students who met the eligibility criteria and voluntarily agreed to participate were included in the study. A total of 69 participants—comprising those who did not wish to participate (n = 62) and those with chronic illnesses (n = 7) were excluded from the study. The participants’ basic demographic characteristics, reasons for choosing their profession, and satisfaction levels were presented in Table 1.

The students’ total score on the QQS was 48.14 ± 9.80. In the lower sections of the scale, “Decreased Commitment” scored 14.61 ± 3.51, “Lack of Motivation” scored 10.54 ± 2.74, “Socialization” scored 11.89 ± 3.66, and “Self-Protection” scored 11.08 ± 2.88. For the MBI-SF dimensions, the mean scores were 14.27 ± 4.66 for Exhaustion, 9.89 ± 3.65 for Cynicism, and 11.28 ± 2.98 for Professional Efficacy (Table 2). In the present sample, the internal consistency coefficients (Cronbach’s α) for the QQS subscales were 0.767 for Decreased Commitment, 0.340 for Lack of Motivation, 0.797 for Socialization, and 0.571 for Self-Protection. For the MBI-SF, Cronbach’s α coefficients were 0.851 for Exhaustion, 0.838 for Cynicism, and 0.636 for Professional Efficacy.

In the comparison of QQS scores by gender, a difference between the groups was found only in the “Self-Protection” subscale (Mann–Whitney U = 4540.00, Z = −1.971, *p* = 0.049, r = 0.131). There were no differences in the other subscales or in the total score (*p* > 0.05). According to the students’ grade levels, significant differences were observed in the scores for Decreased Commitment (H(3) = 15.317, *p* = 0.002, ε^2^ = 0.056), Lack of Motivation (H(3) = 13.584, *p* = 0.004, ε^2^ = 0.048), Self-Protection (H(3) = 16.600, *p* = 0.001, ε^2^ = 0.061), and total scores (H(3) = 17.945, *p* < 0.001, ε^2^ = 0.068). In the socialization subsection, however, no significant difference was found (*p* = 0.234). In the comparison based on students’ reasons for choosing a major, significant differences were observed in the “Decreased Commitment,” (H(3) = 16.290, *p* = 0.001, ε^2^ = 0.060) “Self-Protection,” (H(3) = 11.437, *p* = 0.010, ε^2^ = 0.038) and total scores (H(3) = 15.100, *p* = 0.002, ε^2^ = 0.055). No significant differences were found in the subcategories of “Lack of Motivation” and “Socialization” (*p* = 0.082 and *p* = 0.116, respectively). There were no significant differences between the groups in terms of satisfaction levels across all subdimensions and total scores of the QQS (*p* > 0.05) (Table 3). No significant differences were found in any MBI-SF dimension according to gender, grade level, reason for choosing the programme, or programme satisfaction (*p* > 0.05) (Table 4).

The MBI-SF’s “Exhaustion” and “Cynicism” subscales correlated with the QQS’s “Decreased Commitment” (r = 0.497, r = 0.458, *p* < 0.001, respectively), “Lack of Motivation” (r = 0.430, r = 0.556, *p* < 0.001, respectively), “Socialization” (r = 0.545, r = 0.579, *p* < 0.001, respectively), “Self-Protection” (respectively, r = 0.307, r = 0.374, *p* < 0.001), and “Total” (respectively, r = 0.587, r = 0.639, *p* < 0.001). The MBI-SF “Professional Efficacy” subscale was significantly but weakly correlated with the QQS “Lack of Motivation” subscale (r = 0.143, *p* = 0.032), whereas no significant correlations were observed between “Professional Efficacy” and the other QQS subscales or the QQS total score (*p* > 0.05) (Table 5).

## 4. Discussion

This study investigated the relationship between quiet quitting tendencies and dimensions of burnout among PTR students, as well as differences according to gender, grade level, reasons for choosing the programme and programme satisfaction. The findings showed that PTR students exhibited a moderate level of quiet quitting. Significant differences in quiet quitting were observed according to grade level and reasons for choosing the programme, whereas the only gender-related difference was found in the Self-Protection subscale. No significant differences were observed in the Exhaustion, Cynicism, or Professional Efficacy dimensions of the MBI-SF according to gender, grade level, reason for choosing the programme, or programme satisfaction. Furthermore, Exhaustion and Cynicism were positively associated with the Decreased Commitment, Lack of Motivation, Socialization, and Self-Protection dimensions and with the total QQS score, whereas Professional Efficacy showed only a weak positive association with Lack of Motivation. To our knowledge, this was the first study to examine this association among PTR students, and it highlights the importance of considering both concepts in physiotherapy education.

The demographic characteristics of the students revealed that the mean age was 21.94 ± 2.79 years and that the majority were female. These characteristics are comparable to those reported in previous studies involving health sciences students [19,20]. In the present study, the total QQS score indicated a moderate level of quiet quitting among PTR students (48.14 ± 9.80). Although studies examining quiet quitting among college students are limited, Erol et al. (2024) reported that the level of quiet quitting among undergraduate students was moderate [5]. Similarly, Tok and Uzun (2025) found that the level of quiet quitting was moderate in nursing students [1]. This finding is consistent with previous studies. PTR students may face various psychological and academic challenges due to their intensive academic and clinical training programs [21]. The present findings add to this emerging evidence by indicating that quiet quitting tendencies are also present within physiotherapy education. The moderate level observed in the present study may be considered in relation to reduced voluntary participation in educational activities and lower academic engagement, which are characteristic features of academic quiet quitting. A comparison by gender revealed that male students’ scores on the ‘Self-Protection’ subscale were significantly higher than those of female students. Similarly, studies conducted among both healthcare professionals and nursing students have reported higher QQS scores among male participants, and our findings are consistent with these studies [1,22]. However, since the significant gender difference in the present study was limited to the Self-Protection subscale, this finding should not be generalized to overall quiet quitting. It was reported that female students exhibited higher levels of motivation and engagement in learning compared to male students, while male students showed a more pronounced tendency to disengage from the learning process in evaluating the effect of gender on motivation and engagement in learning among PTR students [23]. A scoping review also reported that men experience a lower sense of belonging at work compared to women and are more likely to engage in quiet quitting [24]. Although this evidence was obtained in a different context, it may provide a relevant perspective for interpreting the gender-related pattern observed in the present study. The present findings do not clarify the factors underlying this difference. From an educational perspective, attention to gender-related differences in specific dimensions of quiet quitting may be useful when monitoring students’ academic engagement and planning supportive educational practices.

Another factor associated with quiet quitting was the level of education. The fourth-year students achieved higher scores on the Decreased Commitment, Lack of Motivation and Total Quiet Quitting measures than the first-year students. The scores of third- and fourth-year students on the Self-Protection subscale were significantly higher than those of first-year students. The final year of the PTR program is a period which students begin to apply their theoretical knowledge in clinical settings and face professional responsibilities more intensively. During this process, students are expected to achieve high academic standards and develop their clinical decision-making, patient management and professional conduct skills [25]. Consistent with this context, Gallasch et al. (2022) reported that students experience substantial challenges during clinical training in relation to high expectations and increased responsibilities [10]. The concentration of higher Decreased Commitment and Lack of Motivation scores among fourth-year students may reflect a stage-specific pattern in which academic expectations, clinical responsibilities, and preparation for professional practice become more prominent. Rather than representing a linear progression of quiet quitting across the years of education, this pattern may indicate that certain dimensions of quiet quitting become more salient at particular stages of PTR training. Another variable associated with quiet quitting was the reason for choosing the programme. Students who chose the programme for reasons such as their admission score or family and social recommendations showed higher quiet quitting scores in some comparisons than those who chose the programme based on personal interest or because it represented a healthcare profession. This pattern may indicate that the extent to which programme choice aligns with students’ interests and professional expectations is relevant to their academic commitment and quiet quitting tendencies. Previous studies have reported that students who consciously and voluntarily choose their careers exhibit higher levels of professional commitment; in parallel, they demonstrate more positive outcomes in terms of academic motivation and engagement in learning [26,27]. Taken together, these findings suggest that the reason for programme choice may have interpretative value beyond a simple background characteristic, as it may reflect the extent to which students’ personal interests and professional expectations align with the educational path they have chosen. In this context, differences in quiet quitting tendencies may be understood as reflecting variations in students’ academic and professional engagement with their chosen field. Accordingly, the reason for programme choice may be particularly relevant when interpreting commitment-related differences in quiet quitting among PTR students.

In the present study, no significant differences were observed in the Exhaustion, Cynicism, or Professional Efficacy dimensions according to gender, grade level, reason for choosing the programme, or programme satisfaction. This pattern suggests that these characteristics alone may be insufficient to account for variation in the burnout dimensions among PTR students. These findings suggest that the expression of burnout among PTR students may extend beyond the demographic and educational characteristics examined in the present study, pointing to the relevance of a broader academic and psychosocial context. Previous studies have linked burnout among physiotherapy students to a range of academic and psychosocial factors [28,29]. In light of this evidence, the absence of significant differences in the present study may indicate that a broader context should be considered when interpreting burnout in PTR students. However, because these factors were not assessed, no direct conclusions can be drawn regarding their relevance to the current findings. Future research including additional contextual variables may provide a more comprehensive understanding of burnout in this population.

Finally, significant associations were observed between quiet quitting and burnout in the present study. Exhaustion and Cynicism were consistently associated with all dimensions of quiet quitting and with the total QQS score, whereas Professional Efficacy showed only a weak association with Lack of Motivation. Previous studies have likewise reported significant relationships between quiet quitting and burnout [30,31]. The different pattern observed across the burnout dimensions is noteworthy. While the consistent associations involving Exhaustion and Cynicism indicate a closer correspondence with quiet quitting, the limited relationship with Professional Efficacy suggests that the strength of this association varies across burnout dimensions. Previous research has also supported the multidimensional structure of student burnout, with Exhaustion, Cynicism, and Professional Efficacy representing separate dimensions that may show different patterns across student profiles [12,32]. In this respect, the present findings suggest that quiet quitting and burnout share some related features, particularly in relation to exhaustion and disengagement, while the degree of association differs across burnout dimensions. Therefore, these findings support considering quiet quitting and burnout as related, but not equivalent, constructs within students’ academic experiences.

### Limitations

This study has some limitations. Firstly, the fact that the scales are self-report measures and that the responses obtained may reflect social desirability should be considered when interpreting the findings. In addition, the very low internal consistency of the Lack of Motivation subscale of the QQS (Cronbach’s α = 0.340) should be taken into account when interpreting findings related to this subscale. Secondly, the fact that the study was conducted on a voluntary basis and that data collection was carried out via Google Forms may have increased the likelihood of participation by individuals with internet access who were interested in the topic, thereby creating a risk of selection bias. Thirdly, conducting the study within a single PTR department may limit the extent to which the findings can be generalized to students at other universities. Finally, the cross-sectional design of the study limits the ability to establish a causal relationship between the variables. Despite all these limitations, this study contributes to understanding the relationship between quiet quitting tendencies and burnout among PTR students and may guide future research examining these issues in the context of PTR education.

## 5. Conclusions

This study demonstrated that quiet quitting is present at a moderate level among PTR students and is significantly associated with the Exhaustion and Cynicism dimensions of burnout. Quiet quitting also differed according to grade level and reason for choosing the programme, while the gender-related difference was confined to the Self-Protection dimension. In contrast, burnout dimensions did not differ according to gender, grade level, reason for choosing the programme, or programme satisfaction. These findings highlight the relevance of considering quiet quitting and burnout together as related but distinct constructs among PTR students. Future multicenter and longitudinal studies may further clarify the relationship between quiet quitting and burnout among PTR students and examine the academic and psychosocial factors associated with these constructs.

## Figures and Tables

**Table 1:** Key Characteristics of Students.

(n = 225)		Mean ± SD	Min–Max
**Age, year**		21.94 ± 2.79	18–45
		**n (%)**	
**Gender**	**Female**	155 (68.9)	
	**Male**	70 (31.1)	
**Grade Level**	**1.** **Grade**	50 (22.2)	
	**2.** **Grade**	56 (24.9)	
	**3.** **Grade**	57 (25.3)	
	**4.** **Grade**	62 (27.6)	
**Reason for choice**	**Being a healthcare profession**	89 (39.6)	
	**Interest in the Profession/Field**	46 (20.4)	
	**Score obtained is sufficient for admission to program**	60 (26.7)	
	**Family/Community Recommendation**	30 (13.3)	
**Level of satisfaction**	**Satisfied**	125 (55.6)	
	**Undecided**	82 (36.4)	
	**Not satisfied**	18 (8)	

SD: Standard deviation, min: Minimum, max: Maximum.

**Table 2:** Students’ Tendencies to Quiet Quitting and Their Levels of Burnout.

(n = 225)		Mean ± SD	Min–Max
**QQS**	Decreased Commitment	14.61 ± 3.51	4–20
	Lack of Motivation	10.54 ± 2.74	5–20
	Socialization	11.89 ± 3.66	4–20
	Self-Protection	11.08 ± 2.88	4–20
	**Total**	48.14 ± 9.80	21–80
**MBI-SF**	Exhaustion	14.27 ± 4.66	5–25
	Cynicism	9.89 ± 3.65	4–20
	Professional Efficacy	11.28 ± 2.98	4–17

SD: Standard deviation, min: Minimum, Max: Maximum, QQS: Quiet Quitting Scale, MBI-SF: Maslach Burnout Inventory—Student Form.

**Table 3:** Comparison of Quiet Quitting Tendencies According to Students’ Characteristics.

		Quiet Quitting Scale				
		Decreased Commitment	Lack of Motivation	Socialization	Self-Protection	Total
**Gender**	**Female**	15 (12–17)	10 (9–12)	12 (9–14)	11 (9–12)	47 (41–53)
	**Male**	15 (12–18)	10.50 (9–13)	12 (9.75–15)	12 (9–14)	49 (42–55.25)
	***p*** **^a^**	0.685	0.307	0.135	**0.049**	0.141
**Grade level**	**1st Grade (1)**	14 (11–15)	10 (8–11)	11 (9–13.25)	10 (8–12)	43 (37–48.25)
	**2nd Grade (2)**	15 (12–18.75)	10 (9–12.75)	13 (9.25–15)	11 (9–13)	49 (41.25–54.75)
	**3rd Grade (3)**	15 (12–17)	10 (8–12)	12 (10–13)	11 (9–13)	47 (43.50–51)
	**4th Grade (4)**	16 (13–18)	11 (9–13.25)	12 (8–16)	12 (10–14)	51 (43.75–57.50)
	***p*** **^b^**	**0.002**	**0.004**	0.234	**0.001**	**<0.001**
	**Comparison between groups (Adjusted *p*)**	**1–4 (0.001)**	**1–4 (0.003)**		**1–3 (0.042)** **1–4 (<0.001)**	**1–2 (0.015)** **1–4 (<0.001)**
**Reason for Choosing Program**	**Being a healthcare profession (1)**	14 (12–17)	10 (8–12)	11 (9–14)	11 (9–12)	45 (41–52)
	**Interest in the Profession/Field (2)**	14 (10–16)	10 (8.75–12)	12 (8–14)	10 (8.75–12)	45 (37–52)
	**Score obtained is sufficient for admission to program (3)**	16 (14–18)	10 (9–12.75)	12 (10–14)	12 (10–14)	49 (44–55)
	**Family/Community Recommendation (4)**	15.50 (12.75–18)	11 (10–12.25)	14 (11.50–15)	12 (9–13.25)	51 (44.75–58.25)
	*p* ^b^	**0.001**	0.082	0.116	**0.010**	**0.002**
	**Comparison between groups (Adjusted *p*)**	**2–3 (0.002)** **1–3 (0.007)**			**2–3 (0.022)**	**2–3 (0.016)** **2–4 (0.043)** **1–3 (0.034)**
**Level of Satisfaction**	**Satisfied**	14 (12–17)	10 (9–12)	12 (9–14)	11 (9–13)	47 (41.50–52.50)
	**Undecided**	15 (12–17)	10 (9–12)	12 (8.75–15)	11 (9–13)	47 (40.75–55)
	**Not satisfied**	17.50 (13.75–18.50)	11.50 (9–14)	13 (9.75–16.50)	12 (10–14.50)	50 (46.25–60.75)
	***p*** **^b^**	0.090	0.125	0.144	0.387	0.108

Data are presented as median (Q1–Q3). ^a^ Mann–Whitney U test. ^b^ Kruskal–Wallis Test. Numbers in the “Comparison between groups” row indicate the grade/category groups that differed significantly in post hoc pairwise comparisons, and adjusted *p*-values are reported. Statistical significance was set at *p* < 0.05.

**Table 4:** A Comparison of Students’ Burnout Levels Based on Key Characteristics.

		MBI-SF		
		Exhaustion	Cynicism	Professional Efficacy
**Gender**	**Female**	14 (11–18)	9 (7–12)	12 (9–13)
	**Male**	12.50 (10–17.25)	9 (7–12)	11 (9–14)
	***p*** **^a^**	0.563	0.663	0.950
**Grade Level**	**1st Grade (1)**	12 (10–16)	8 (7.75–10.25)	12 (10–14)
	**2nd Grade (2)**	14.50 (11–19)	10 (7.25–12.75)	11 (8–13)
	**3rd Grade (3)**	12 (10–18.50)	9 (7–12)	12 (10–13)
	**4th Grade (4)**	14 (10.75–19.25)	9 (7–14)	11 (9–13)
	***p*** **^b^**	0.212	0.447	0.067
**Reason for Choosing Program**	**Being a healthcare professional (1)**	12 (10–16)	9 (7–11)	11 (9–13)
	**Interest in the Profession/Field (2)**	12.50 (10.75–17.25)	8 (7.75–11.25)	11 (8–13)
	**Score obtained is sufficient for admission to program (3)**	15 (12–18.75)	10 (8–13.75)	11.50 (9–13)
	**Family/Community Recommendation (4)**	13.50 (10–19.25)	9 (8–12)	13 (10.75–15)
	*p* ^b^	0.080	0.119	0.084
**Level of satisfaction**	**Satisfied**	12 (10–17)	8 (7–11)	12 (9–13)
	**Undecided**	14 (11–19)	9 (7–13)	11 (9–13)
	**Unsatisfied**	13.50 (10.75–18.50)	10.50 (8–14.25)	12 (9.75–12)
	***p*** **^b^**	0.388	0.135	0.907

Data are presented as median (Q1–Q3). ^a^ Mann–Whitney U test. ^b^ Kruskal–Wallis Test, MBI-SF: Maslach Burnout Inventory—Student Form. Statistical significance was set at *p* < 0.05.

**Table 5:** The Relationship Between Students’ Quiet Quitting Tendencies and Burnout Dimensions.

			Quiet Quitting Scale				
			Decreased Commitment	Lack of Motivation	Socialization	Self-Protection	Total
**MBI-SF**	Exhaustion	r	0.497	0.430	0.545	0.307	0.587
		*p*	**<0.001**	**<0.001**	**<0.001**	**<0.001**	**<0.001**
	Cynicism	r	0.458	0.556	0.579	0.374	0.639
		*p*	**<0.001**	**<0.001**	**<0.001**	**<0.001**	**<0.001**
	Professional Efficacy	r	−0.110	0.143	0.084	−0.023	0.062
		*p*	0.100	**0.032**	0.212	0.734	0.354

MBI-SF: Maslach Burnout Inventory—Student Form. Spearman correlation test.

## Data Availability

The data are available from the corresponding author upon reasonable request due to privacy and ethical restrictions.
